# Cynaropicrin Suppresses Cell Proliferation by Inducing Mitophagy through p38 MAPK-Mediated Mitochondrial ROS Generation in Human Hepatocellular Carcinoma Cells

**DOI:** 10.4014/jmb.2501.01025

**Published:** 2025-04-24

**Authors:** Min Yeong Kim, Hyun Hwangbo, Seon Yeong Ji, Da Hye Kim, Shin-Hyung Park, Su Hyun Hong, Gi Young Kim, EunJin Bang, Yung Hyun Choi

**Affiliations:** 1 Basic Research Laboratory for the Regulation of Microplastic-Mediated Diseases and Anti-Aging Research Center, Dong-eui University, Busan 47340, Republic of Korea; 2 Department of Biochemistry, Dong-eui University College of Korean Medicine, Busan 47227, Republic of Korea; 3 Department of Pathology, Dong-eui University College of Korean Medicine, Busan 47227, Republic of Korea; 4 Laboratory of Immunobiology, Department of Marine Life Sciences, Jeju National University, Jeju 63243, Republic of Korea

**Keywords:** Cynaropicrin, hepatocellular carcinoma cells, mitophagy, reactive oxygen species, p38 MAPK

## Abstract

Cynaropicrin, a sesquiterpene lactone, has diverse pharmacological activities. However, its anticancer activity against hepatocellular carcinoma (HCC) has not been fully elucidated. Here, we investigated the cytotoxic effects of cynaropicrin and examined its mechanism of action in human HCC cells. The results demonstrated that cynaropicrin significantly induced cytotoxicity and autophagy in HCC cells, but not in immortalized non-cancerous hepatocytes, which was related to the generation of mitochondrial reactive oxygen species (mtROS) and induction of mitochondrial membrane potential loss. Under cynaropicrin treatment, the expression of microtubule-associated protein light chain 3, which is involved in the elongation of the phagophore membrane, was upregulated, whereas the expression of Beclin-1 and p62, which are essential for the formation of autophagosomes, was downregulated. In addition, the expression of mitophagy regulators PTE*N*-induced kinase 1 (PINK1) and Parkin in the mitochondria increased, suggesting the induction of autophagic flux in the mitochondria. However, *N*-acetyl-l-cysteine, a ROS scavenger, counteracted cynaropicrin-induced effects. Moreover, cynaropicrin increased the phosphorylation of p38 mitogen-activated protein kinase (MAPK), and the p38 MAPK inhibitor, SB203580, specifically attenuated cynaropicrin-induced cytotoxicity and mtROS production. Importantly, SB203580 reversed cynaropicrin-induced expression of PINK1 and Parkin in the mitochondria. Collectively, our findings demonstrate that cynaropicrin exerts cytotoxic effects against HCC cells by inducing mitochondrial autophagy through the activation of the p38 MAPK-ROS pathway, indicating that cynaropicrin could be a potential therapeutic agent for liver cancer treatment.

## Introduction

Hepatocellular carcinoma (HCC) is a frequently diagnosed common type of primary liver cancer that has a high mortality rate and is the second most frequent cause of cancer-related deaths [1, 2]. Primary risk factors for HCC include chronic liver disease, liver cirrhosis, and hepatitis virus infection [3, 4]. However, effective treatment of HCC remains elusive because of its ineffectiveness and high recurrence rates. Therefore, discovery of new candidates for liver cancer prevention and treatment is urgently required. Interest in approaches that use natural products that are not harmful to normal cells has recently increased.

Cynaropicrin is a sesquiterpene lactone isolated from artichoke (*Cynara scolymus* L.) [5]. The beneficial effects of cynaropicrin in various human disease models have been reported to be based on its antioxidant and anti-inflammatory effects [6-9]. Additionally, cynaropicrin exerts cytotoxic effects in multiple cancer cell lines. The mechanisms of action of these anticancer effects include the induction of apoptosis and cell cycle arrest [10-17]. Furthermore, the cytotoxic effects of cynaropicrin in leukemia cells appear to be mediated by reactive oxygen species (ROS) [18]. These results support the finding that cynaropicrin exerts cytotoxic effects by inducing oxidative stress in human glioblastoma and cervical carcinoma cells [12, 16]. Although there are multiple sources of ROS within cells, mitochondria are the central organelles that produce them, and excessive ROS levels can cause cell damage and death [5, 19].

Many intracellular signaling pathways are involved in cancer cell proliferation. Among them, mitogen-activated protein kinases (MAPKs), including c-Jun *N*-terminal kinase (JNK), extracellular signal-regulated kinase (ERK), and p38 MAPK, are altered in several types of cancer, and pharmacologically selective inhibition of MAPKs has become a candidate target for tumor treatment [20, 21]. Previous studies have shown that MAPKs are activated or inhibited by various natural products with anticancer activity, contributing to the inhibition of various cancer phenotypes such as cell proliferation, invasion, and metastasis [22, 23]. MAPKs are also involved in the anticancer activity of cynaropicrin. For example, in lymphoma cells, cynaropicrin inhibited the activity of major adhesion molecules by interfering with ERK activation, leading to cell cycle arrest and promotion of apoptosis [18]. In addition, inactivation of ERK has been reported to accompany cynaropicrin treatment-induced apoptosis in melanoma and glioma cells [12, 15], but changes in the activity of other major kinases belonging to MAPKs, such as p38 or JNK, have not been reported to date.

Autophagy, which inhibits or promotes apoptosis, has recently been identified as one of the main mechanisms underlying several types of cell death [24, 25]. In particular, the balance between autophagy and apoptosis is important for regulating hepatocyte turnover and maintaining intracellular homeostasis [26], and an imbalance between the two is commonly observed in many cancers, including HCC. According to Yang *et al*. [11], the induction of apoptosis by cynaropicrin in neuroblastoma cells is accompanied by endoplasmic reticulum (ER) stress-mediated autophagy, which contributes to apoptosis inhibition. Autophagy is closely associated with ER stress and mitochondrial dysfunction [24, 27]. Recently, Boulos *et al*. [10] proposed that cynaropicrin exerts cytotoxicity in multiple myeloma cells by inducing parthanatos following the translocation of apoptosis-inducing factors present in the mitochondrial intermembrane space to the nucleus. In addition, Rotondo *et al*. [12] suggested that the activation of apoptosis and autophagy by cynaropicrin in human glioblastoma cells was achieved by inducing cytochrome c release following mitochondrial membrane potential (MMP) loss, which causes ROS generation. These findings demonstrated that mitochondrial damage plays a central role in the anticancer activity of cynaropicrin. However, the link between ROS, autophagy, apoptosis, in the anticancer activity of cynaropicrin remains unclear. In particular, there has been no report on the involvement of mitophagy, the selective degradation of mitochondria through autophagy, in cynaropicrin-induced apoptosis not only in HCC cells but also in other cancer cell lines. Therefore, in this study, we investigated the potential anticancer effects of cynaropicrin and its mechanism of action in HCC cells, particularly the role of ROS in the regulation of mitochondrial autophagy.

## Materials and Methods

### Cell Culture and Treatment

Human HCC cell lines (Hep3B and HepG2) and normal adult liver epithelial cells (THLE-2) were obtained from American Type Culture Collection (ATCC, USA). All cells were cultured in Dulbecco’s modified Eagle’s medium supplemented with 10% fetal bovine serum and 100 U/ml penicillin/streptomycin. Cells were cultured at 37°C and 5% CO_2_. The materials required for cell culture were purchased from WelGENE, Inc., (Republic of Korea). To investigate the effect of cynaropicrin (Sigma-Aldrich, USA) on cell proliferation, the cells were treated with various concentrations of cynaropicrin and cultured for 24 h. In addition, to evaluate the effect of cynaropicrin on intracellular signaling systems, cells were pretreated with bafilomycin A1, *N*-acetyl-L-cysteine (NAC), Mito-TEMPO, and MAPK inhibitors (Sigma-Aldrich) for 1 h prior to cynaropicrin treatment.

### Cell Viability and Apoptosis Detection

Cell viability was measured using the 3-[4,5-dimethylthiazol-2-yl]-2,5-diphenyltetrazolium bromide (MTT) assay, as previously described [28]. Briefly, 0.5 mg/mL MTT solution (Thermo Fisher Scientific, USA) was added to the cells cultured under various conditions and reacted for 2 h. The culture medium was then removed and dimethyl sulfoxide (Sigma-Aldrich) was added to each well to dissolve the formazan. Cell viability was determined by measuring absorbance at 540 nm using a microplate reader (VERSA Max, Molecular Devices, USA). To confirm that cynaropicrin induced apoptosis, a commercially available terminal deoxynucleotidyl transferase dUTP nick end labeling (TUNEL) Staining Assay Kit (Abcam, UK) was used. Briefly, cynaropicrin-treated cells were fixed with 4% paraformaldehyde (Sigma-Aldrich) and permeabilized with 0.3% Triton X-100 (Sigma-Aldrich). Cells were stained with TUNEL detection reagent for 2 h, DNA was counterstained with 4',6-diamidino-2-phenylindole (DAPI, Thermo Fisher Scientific) and propidium iodide (Sigma-Aldrich), and images were acquired using a fluorescence microscope (EVOS FS Auto; Thermo Fisher Scientific). Changes in cell morphology following cynaropicrin treatment were observed using a phase-contrast microscope (Carl Zeiss, Germany).

### Flow Cytometry Analysis

To determine whether autophagy was induced, cells were stained using the CYTO-ID Autophagy Detection Kit (Enzo Life Sciences, USA), according to the manufacturer's protocol [29]. Briefly, cells were washed with phosphate-buffered saline (PBS), treated with CYTO-ID Green detection reagent and incubated for 30 min in the dark. Autophagy induction was measured using flow cytometry (BD Biosciences, USA). The experimental procedures used to investigate cynaropicrin-induced MMP changes followed those described in previous studies [30]. The cells were incubated with 5,5',6,6'-tetrachloro-1,1',3,3'-tetraethylbenzimidazolyl-carbocyanine iodide (JC-1, Abcam) for 20 min in the dark, the pellet was resuspended in PBS, and MMP changes were examined using flow cytometry. Additionally, 2',7'-dichlorodihydrofluorescein diacetate (DCF-DA; Thermo Fisher Scientific) and MitoSOX (Invitrogen, USA) were used to detect intracellular ROS and mitochondrial reactive oxygen species (mtROS) production. The treated cells were collected, incubated with DCF-DA and MitoSOX dye for 20 min, and used for flow cytometry according to the manufacturer details.

### Immunofluorescence

For immunofluorescence analysis, cells cultured under various conditions were attached to curved slips using a Thermo Shandon Cytospin 3 Cell Centrifuge (Marshall Scientific, USA). To confirm autophagy induction by fluorescence microscopy, the nuclei of cells stained with CYTO-ID Green detection reagent were counterstained with DAPI. After staining, the cells were fixed with 4% paraformaldehyde, washed with PBS, and fluorescence intensity was observed under a fluorescence microscope [31]. The fluorescence intensities of JC-1 monomers and JC-1 aggregates in cells stained with JC-1 were compared under a fluorescence microscope to observe the changes in MMP. To detect autophagosome formation using microtubule-associated protein light chain 3 (LC3), cells were fixed with 100% methanol and blocked with blocking buffer [5% bovine serum albumin (BSA) in PBS-T (0.1%Triton X)] for 1 h at room temperature (RT). After fixation, the cells were incubated with an anti-LC3 antibody diluted in 2.5% BSA overnight at 4°C. The production of intracellular ROS and mtROS was also observed by DCF-DA and MitoSOX staining, and additional staining was performed using DAPI or MitoTracker (Thermo Fisher Scientific) to stain the nucleus and mitochondria [32].

### Western Blotting

Cells treated with cynaropicrin for 24 h in the presence or absence of bafilomycin A1, NAC, or the p38 MAPK inhibitor (SB203580) were collected. Cells were suspended in lysis buffer and incubated at 4°C for 30 min a total protein was extracted as previously described [33]. Mitochondrial and cytosolic proteins were isolated and extracted using the Mitochondrial Isolation Kit (Thermo Fisher Scientific). The protein concentration was measured using a Bio-Rad protein assay (Bio-Rad, USA). Equal amounts of protein were electrophoresed on sodium dodecyl sulfate-polyacrylamide gels and transferred onto nitrocellulose membranes (GE Healthcare, USA). The membrane was blocked with 5% skim milk for 30 min, incubated with appropriate primary antibodies (Santa Cruz Biotechnology Inc., USA; Cell Signaling Technology, USA; and Abcam), and incubated overnight at 4°C. The membrane was washed three times with PBS-T (PBS with Tween 20) for 10 min. The membrane was incubated with secondary horseradish peroxidase-conjugated antibodies (Santa Cruz Biotechnology) for 1 h at RT. After washing the membrane with PBS-T three times for 10 min, protein expression was detected using an enhanced chemiluminescence solution (Thermo Fisher Scientific) and a Fusion Solo S system (Vilber Lourmat, France).

### Statistical Analysis

The results are presented as the mean ± standard deviation. Statistical analyses were performed using one-way ANOVA and Tukey’s post-hoc test using GraphPad Prism version 8.4.2 (GraphPad Software Inc., USA). Statistical significance was set at *p* < 0.05.

## Results

### Cynaropicrin Increased Cell Death and Autophagy Induction

**Fig. 1:**
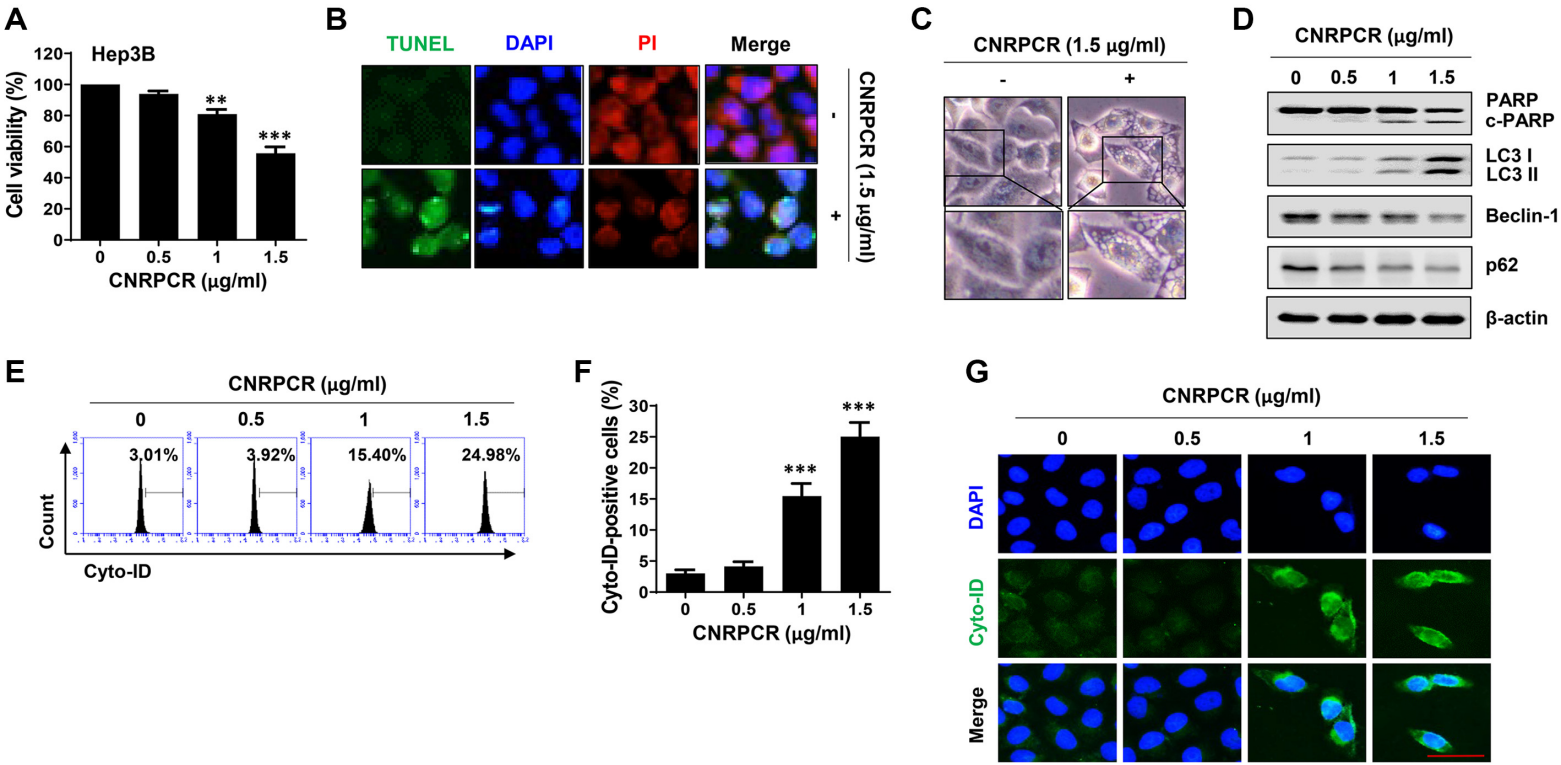
Induction of cytotoxicity and autophagy by cynaropicrin in Hep3B cells. Cells were grown for 24 h in medium containing various doses of cynaropicrin. (**A**) Cell viability was measured using the MTT assay. (**B**) Cells were stained with terminal deoxynucleotidyl transferase dUTP nick end labeling (TUNEL) and propidium iodide (PI) and images were observed under a fluorescence microscope. (**C**) Autophagic vacuoles were detected using phase contrast microscopy. (**D**) Protein expression levels of PARP and autophagy-related molecules (LC3, Beclin-1, and p62) were assessed by Western blotting. (**E**–**G**) Autophagy induction was measured by flow cytometry (**E**) and immunofluorescence (**G**) using Cyto-ID dye. (**F**) Flow cytometry results were quantified and presented as bar graphs (***p < 0.001 vs. untreated group).

The MTT assay was conducted to assess the cytotoxic effects of cynaropicrin on Hep3B cells. As shown in Fig. 1A, cynaropicrin treatment significantly reduced cell viability in a concentration-dependent manner. The cytotoxic effect following cynaropicrin treatment was similarly observed in HepG2 cells, another HCC cell line, but was significantly lower in THLE-2 cells, an immortalized human adult liver epithelial cell line (Supplementary results). Therefore, we investigated whether the inhibitory effect of cynaropicrin on cell survival was related to the induction of apoptosis, using TUNEL staining and Western blotting. As shown in Fig. 1B and 1D, the number of cells positive for TUNEL staining (green) was significantly higher than that in the control group, and cleavage of the DNA repair enzyme poly (ADP-ribose) polymerase (PARP) was induced, indicating that apoptosis was induced by cynaropicrin treatment. In addition, cynaropicrin significantly increased the expression of LC3, a marker protein for autophagy induction, and the conversion of LC3 I to LC3 II, with the formation of autophagic vacuoles in the cytoplasm (Fig. 1C and 1D). Cynaropicrin also notably increased the number of cyto-ID-stained autophagic vacuoles and autophagy flux (Fig. 1E-1G). In contrast, the expression levels of Beclin-1 and p62 notably decreased (Fig. 1D). The cleavage of PARP, induction of autophagy, and increase in LC3 II expression by cynaropicrin treatment were similarly observed in HepG2 cells, but not in THLE-2 cells (Supplementary results).

### Cynaropicrin Promoted Mitochondrial Dysfunction

**Fig. 2:**
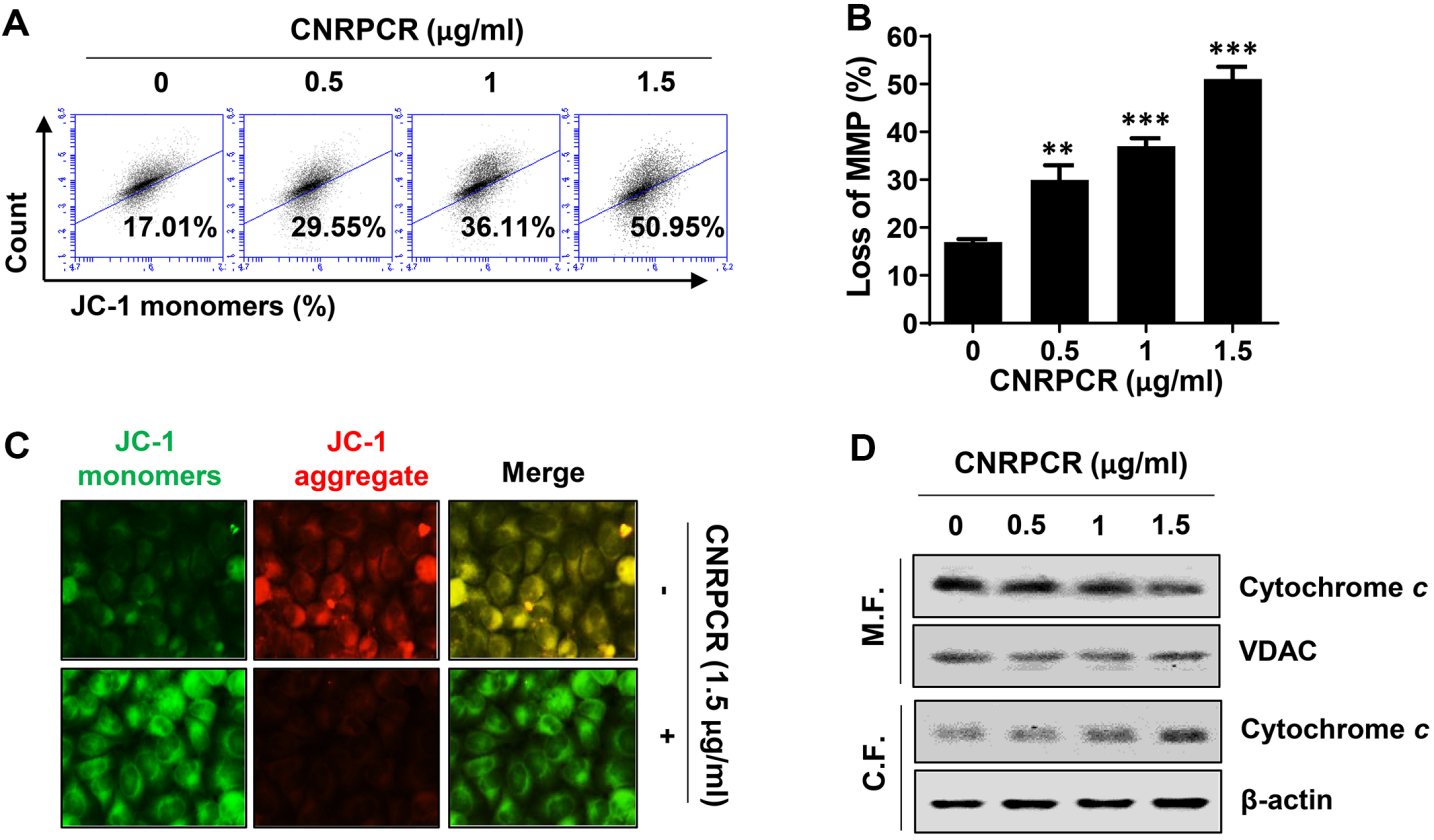
Induction of mitochondrial dysfunction by cynaropicrin in Hep3B cells. Cells were grown for 24 h in medium containing the indicated doses of cynaropicrin. (**A**–**C**) MMP was assessed by flow cytometry and immunofluorescence using the JC-1 dye. (**A**) Mitochondrial function was assessed by flow cytometry using JC-1 dye and the frequency of cells with JC-1 monomers, indicating MMP loss, was quantified. (**B**) Numerical data are shown as mean ± SD (**p < 0.01, ***p < 0.001 vs. untreated group). (**C**) Mitochondrial depolarization by JC-1 staining was compared using the immunofluorescence intensity of JC-1 monomer (green) and JC-1 aggregate (red). (**D**) Expression of cytochrome c was examined Western blotting in the mitochondrial (M.F.) and cytosolic fractions (C.F.).

Next, we evaluated whether cynaropicrin cytotoxicity was associated with the induction of mitochondrial dysfunction. As shown in Fig. 2A and 2B, flow cytometry results demonstrated that the proportion of JC-1 monomers significantly increased with increasing cynaropicrin treatment concentrations, indicating increased mitochondrial membrane permeability and loss of electrical potential. In good agreement with these results, in fluorescence microscopy observation, the intensity of red fluorescence, indicating JC-1 aggregates, increased in cynaropicrin-treated cells compared to the control group, while the green fluorescence intensity, representing JC-1 monomers, was decreased (Fig. 2C). Furthermore, cynaropicrin promoted the release of cytochrome *c* from mitochondria into the cytoplasm in a concentration-dependent manner (Fig. 2D).

### Cynaropicrin Enhanced Mitochondrial Autophagy

**Fig. 3:**
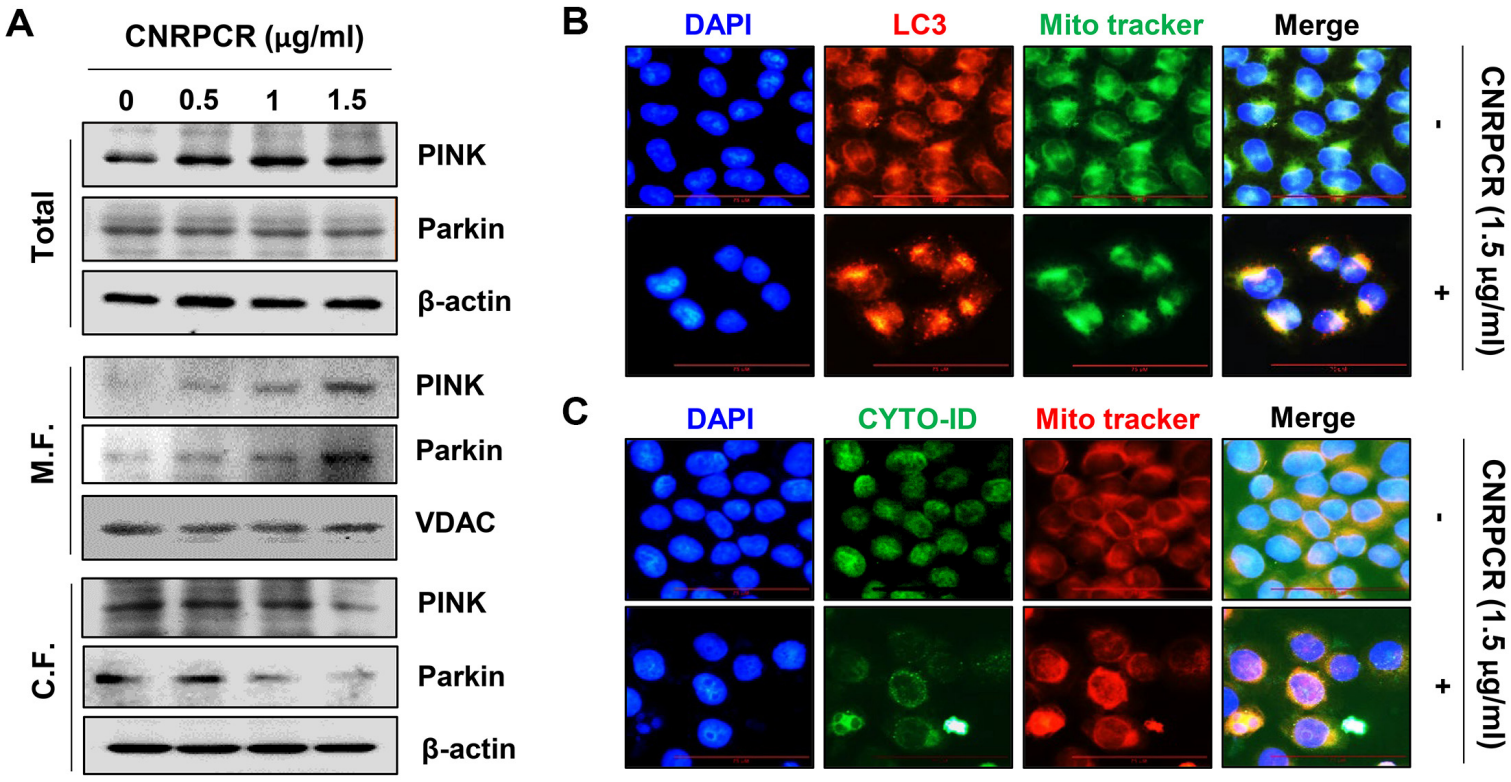
Induction of mitophagy by cynaropicrin in Hep3B cells. Cells were cultured for 24 h in medium containing the indicated doses of cynaropicrin. (**A**) Mitophagy-related proteins PINK1 and Parkin were evaluated in mitochondrial, cytoplasmic, and total protein fractions using Western blotting. (**B**) After staining with LC3 antibody (red) and MitoTracker dye (green), the fluorescence intensity was observed under a fluorescence microscope. 4',6-diamidino-2-phenylindole (DAPI) was used to counterstain the nucleus. (**C**) Images of cells stained with Cyto-ID (green) and MitoTracker (red) were acquired under a fluorescence microscope to detect mitochondrial autophagic vacuole formation.

To investigate whether cynaropicrin promotes mitophagy induced by dysfunctional mitochondria in HCC cells, we performed Western blotting to evaluate the expression of the mitophagy marker proteins PTE*N*-induced kinase 1 (PINK) and Parkin. As shown in Fig. 3A, the protein expression levels of PINK1 and Parkin in Hep3B cells increased in a concentration-dependent manner in the mitochondrial fraction; however, the whole fraction showed no change in these marker proteins. This recruitment of PINK1 and Parkin to mitochondria induced by cynaropicrin was similarly observed in HepG2 cells (Supplementary results). In contrast, they decreased in the cytoplasmic fraction of cells treated with cynaropicrin. To confirm this, an immunofluorescence co-staining assay was conducted to observe the distribution and expression levels of LC3 and MitoTracker, a mitochondrial labeling dye. As shown in Fig. 3B, LC3 colocalized with MitoTracker-stained mitochondria in Hep3B cells treated with cynaropicrin. In addition, autophagic vacuoles stained with Cyto-ID green dye colocalized with MitoTracker after cynaropicrin treatment, suggesting that autophagosomes were induced in the mitochondria (Fig. 3C), indicating that cynaropicrin treatment induced mitochondrial autophagy in HCC cells.

### Cynaropicrin Induced Autophagy during Apoptosis

**Fig. 4:**
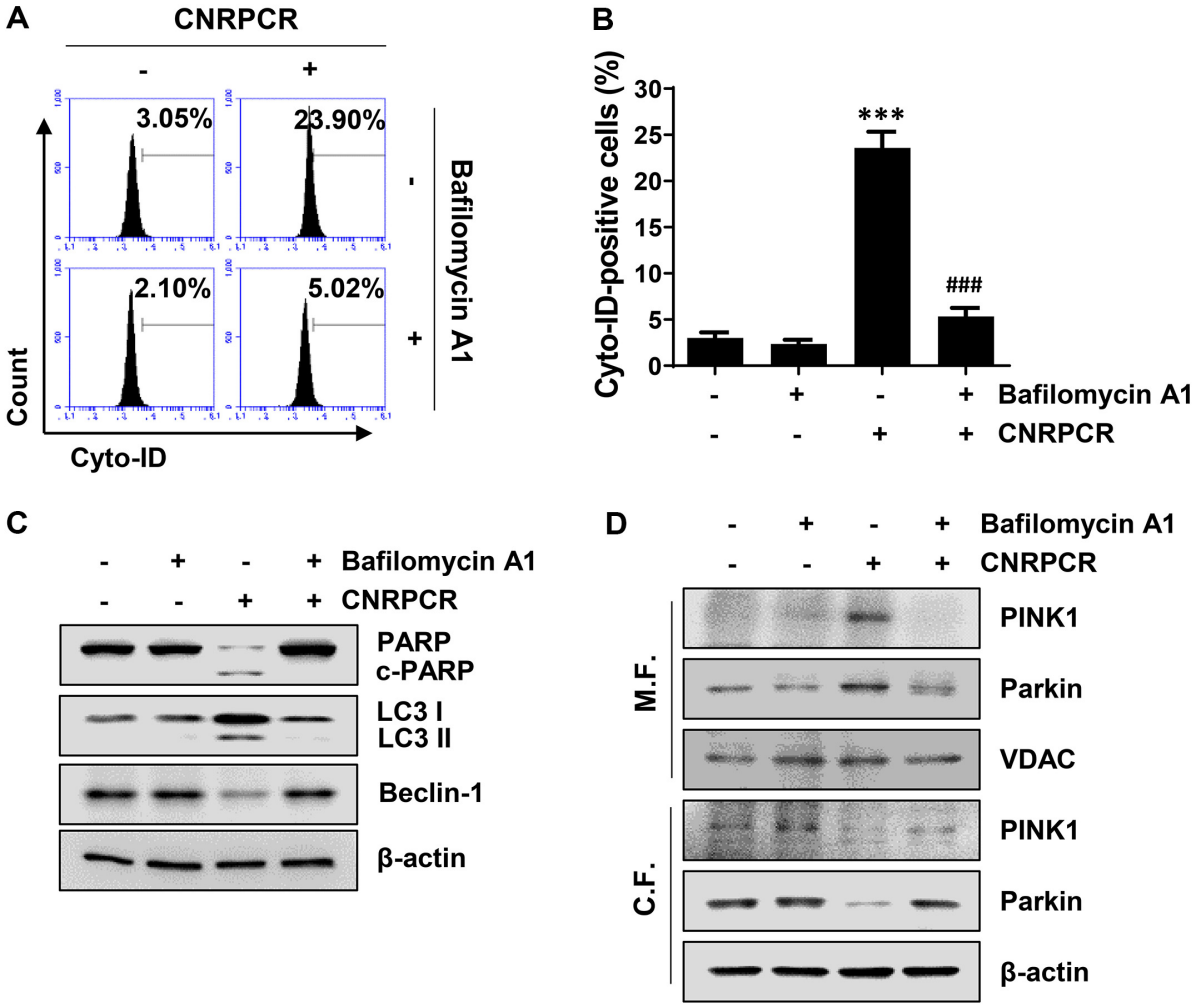
Attenuation of cynaropicrin-induced autophagy and mitophagy by bafilomycin A1 in Hep3B cells. Cells were pretreated with bafilomycin A1 (1 nM) for 1 h and then grown for 24 h in a medium containing cynaropicrin (1.5 μg/ml). (**A**) The extent of autophagy induction was measured by flow cytometry using Cyto-ID. (**B**) Flow cytometry results were quantified and are displayed as bar graphs (***p < 0.001 vs. untreated group; ^###^*p* < 0.001 vs. cynaropicrin-treated group). (**C** and **D**) The expression of the indicated proteins in the total protein (**C**), mitochondrial, and cytosolic fractions (**D**) was analyzed by Western blotting using the corresponding antibodies.

To investigate the role of autophagy in cynaropicrin-induced apoptosis in HCC cells, the cells were pretreated with bafilomycin A1, an autophagy inhibitor, for 1 h, followed by cynaropicrin treatment for 24 h. Flow cytometry analysis showed that the number of cyto-ID-stained autophagosome vacuoles in cells pretreated with bafilomycin A1 was significantly reduced compared with that in cells treated with cynaropicrin alone (Fig. 4A and 4B). According to the Western blotting results (Fig. 4C), bafilomycin A1reduced cynaropicrin-induced cleavage of PARP. In addition, the expression of LC3 and its conversion to LC3 II were significantly abolished by bafilomycin A1, whereas the reduced expression of Beclin-1 by cynaropicrin treatment was maintained at the control level in the presence of bafilomycin A1 (Fig. 4C), suggesting blockage of cynaropicrin-induced autophagy flux. To further assess the role of mitophagy in cynaropicrin-induced apoptosis, we measured the expression of the mitophagy marker proteins. As shown in Fig. 4D, in the mitochondrial fraction of cells pretreated with bafilomycin A1, the increased expression of the mitophagy markers PINK1 and Parkin was significantly reduced compared with that in cynaropicrin-treated cells. In contrast, in the cytoplasmic fraction, bafilomycin A1 increased mitophagy markers PINK1 and Parkin expression, which were reduced by cynaropicrin (Fig. 4D), indicating involvement of mitophagy in cynaropicrin-induced apoptosis in HCC cells.

### ROS Modulated Cynaropicrin-Induced Autophagic Apoptosis

**Fig. 5:**
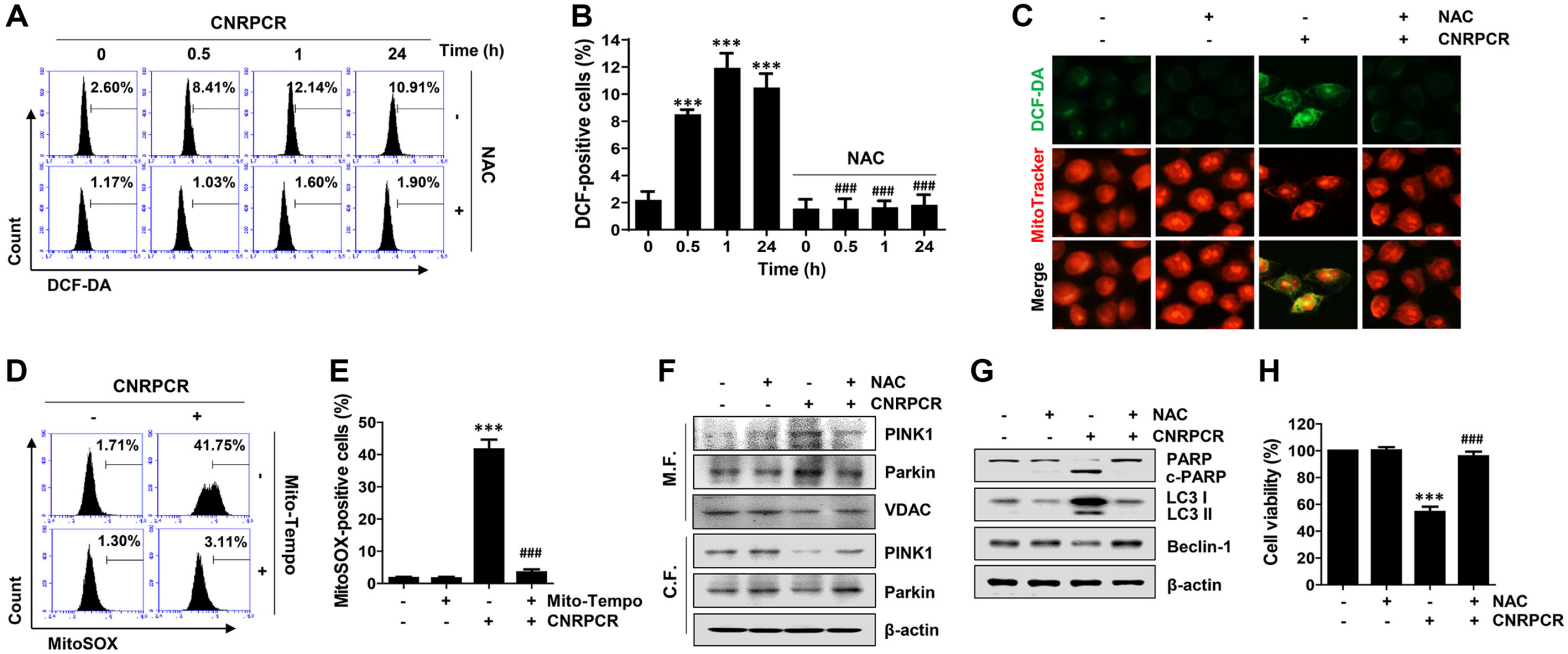
Role of ROS in cynaropicrin-induced autophagy, mitophagy, and cytotoxicity in Hep3B cells. Cells were pretreated with NAC (40 mM) (**A**–**C** and **F**–**H**) or Mito-TEMPO (2 μM) (**D** and **E**) for 1 h. After pretreatment, cells were grown for the indicated times (**A** and **B**), 1 h (**C**–**E**), or 24 h in a medium containing cynaropicrin (1.5 μg/ml). (**A** and **B**) Intracellular ROS levels were detected using flow cytometry with DCF-DA staining (**A**), and DCF-DA-positive cells were quantified and are shown in bar graphs (**B**). (**C**) Cells were co-stained using DCF-DA (green) and MitoTracker (red), and fluorescence images were observed under a fluorescence microscope. (**D** and **E**) Mitochondrial superoxide levels were examined using flow cytometry with MitoSOX staining (**D**), and MitoSOXpositive cells were quantified (**E**). (**E** and **F**) The expression of the indicated proteins in mitochondria, cytosolic fractions (**F**), and total proteins (**G**) was analyzed by Western blotting using the corresponding antibodies. (**H**) Cell viability was measured using the MTT assay. Numerical data are shown as the mean ± SD (***p < 0.001 vs. untreated group; ^###^*p* < 0.001 vs. cynaropicrin-treated group).

Next, we examined whether ROS, which modulate autophagic activity by activating various signaling pathways, are upstream regulators of cynaropicrin-induced mitophagy in HCC cells. Flow cytometric analysis using DCF-DA staining, which reflects intracellular ROS production, demonstrated that cynaropicrin induced a marked increase in intracellular ROS production, which was remarkably reversed by pretreatment with the ROS scavenger NAC (Fig. 5A and 5B). Consistent with the flow cytometry results, cynaropicrin increased the DCF-DA staining intensity, and this effect was reduced by NAC pretreatment (Fig. 5C). Similarly, cynaropicrin markedly increased mitochondrial superoxide levels, as detected by MitoSOX, which were alleviated in the presence of Mito-TEMPO, a mitochondria-targeted antioxidant, to levels comparable to those in the control conditions (Fig. 5D and 5E). Next, we assessed whether ROS generation is involved in the regulation of cynaropicrin-induced autophagy in Hep3B cells. When cells were pretreated with NAC, the expression levels of PINK1 and Parkin decreased in the mitochondrial fraction, suggesting that ROS modulated cynaropicrin-induced mitophagy (Fig. 5F). Furthermore, when the expression changes of PARP, LC3, and Beclin-1 were examined under the same conditions, the cynaropicrin-induced alteration of these proteins was reversed by NAC pretreatment, indicating that ROS modified cynaropicrin-induced autophagy in Hep3B cells. In addition, NAC pretreatment ameliorated cynaropicrin-induced cytotoxicity, demonstrating the critical role of ROS in cynaropicrin-induced autophagic apoptosis in Hep3B cells (Fig. 5H).

### p38 MAPK Attenuated Cynaropicrin-Induced ROS Generation and Cell Death

**Fig. 6:**
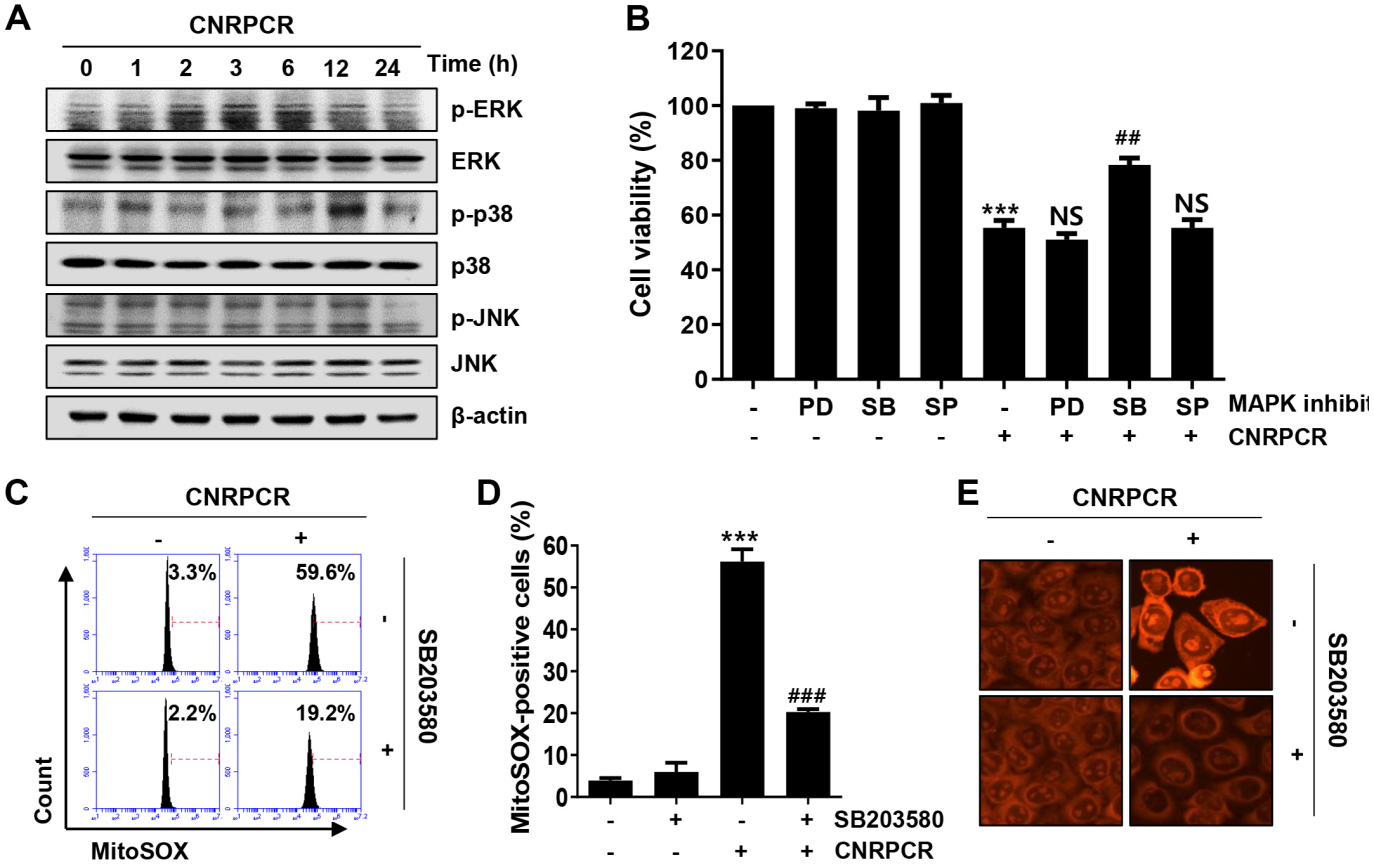
Role of p38 MAPK in cynaropicrin-induced mitochondrial ROS in Hep3B cells. Cells were treated with cynaropicrin for the indicated times (**A**) or pretreated with either MAPK inhibitors, PD (PD98059, ERK inhibitor), SB (SB203580, p38 MAPK inhibitor) or SP (SP600125, JNK inhibitor), for 1 h and then stimulated with cynaropicrin (1.5 μg/ml) for 24 h (**B**) or 1 h (**C**–**E**). (**A**) Protein expression levels of phosphorylated or total ERK, p38 MAPK, and JNK were assessed using Western blotting. (**B**) Cell viability was measured using the MTT assay. (**C**–**E**) The levels of mitochondrial superoxide were examined using flow cytometry with MitoSOX staining (**C**), and MitoSOX-positive cells were quantified (**D**). Additionally, the cells were stained with MitoSOX, and the intensity of mitochondrial superoxide was observed using immunofluorescence. Numerical data are shown as the mean ± SD (***p < 0.001 vs. untreated group; ^##^*p* < 0.01, ^###^*p* < 0.001 vs. cynaropicrin-treated group; NS, not significant).

We investigated the potential role of MAPKs in cynaropicrin-induced autophagy. As shown in Fig. 6A, cynaropicrin increased the phosphorylation levels of ERK and p38 MAPK, starting at 2 and 12 h of treatment, respectively, without significant changes in JNK. To investigate the role of MAPKs in cynaropicrin-induced cytotoxicity, cell viability was examined after treatment with cynaropicrin in the presence or absence of the MAPKs inhibitors, PD98059 (ERK inhibitor), SB203580 (p38 MAPK inhibitor), and SP600125 (JNK inhibitor). The results demonstrated that SB203580 pretreatment restored cynaropicrin-induced cytotoxicity in HCC cells (Fig. 6B and Supplementary results). Furthermore, SB203580 pretreatment notably suppressed mtROS production, which was elevated by cynaropicrin (Fig. 6C-6E), suggesting that p38 MAPK is an upstream regulator of ROS-mediated cynaropicrin-induced cytotoxicity in HCC cells.

### p38 MAPK Modulated Cynaropicrin-Induced Autophagy

**Fig. 7:**
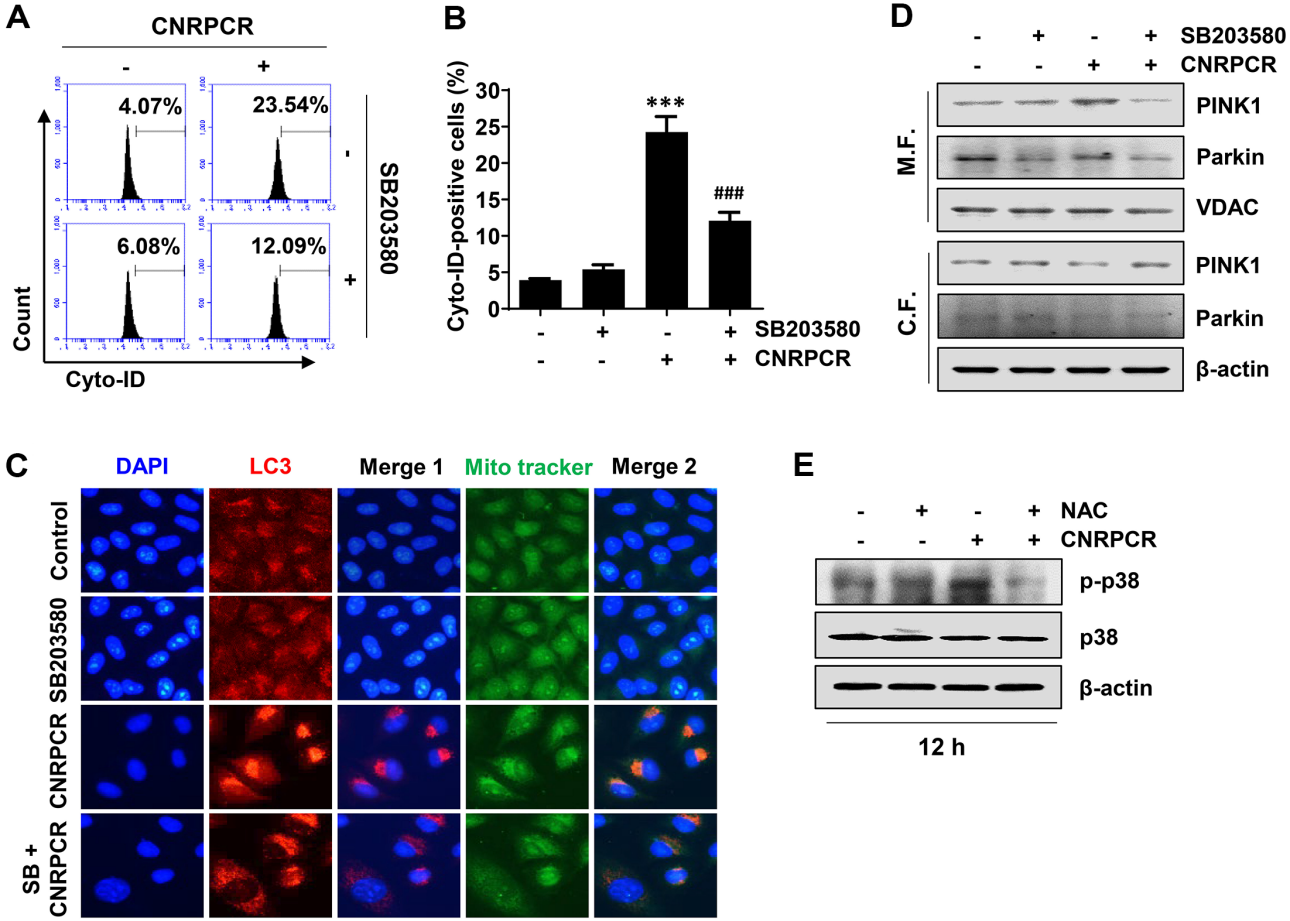
Role of p38 MAPK in autophagy induced by cynaropicrin in Hep3B cells. Cells were treated with the p38 MAPK inhibitor (SB203580) or NAC for 1 h and then stimulated with cynaropicrin for 24 h. (**A** and **B**) The extent of autophagy induction was measured (**A**) and quantified (**B**) by flow cytometry using Cyto-ID (***p < 0.001 vs. untreated group; ^###^*p* < 0.001 vs. cynaropicrin-treated group). (**C**) After staining with LC3 antibody (red) and MitoTracker dye (green), fluorescence images were acquired using fluorescence microscopy. (**D** and **E**) The expression of the indicated proteins in mitochondria, cytosolic fractions (**D**), and total protein (**E**) was analyzed by Western blotting using the corresponding antibodies.

To evaluate the role of p38 MAPK in cynaropicrin-induced autophagy, a downstream signaling cascade of ROS under cynaropicrin, we pretreated cells with SB203580 before cynaropicrin treatment and assessed autophagy and mitophagy-related marker proteins. Flow cytometry using the Cyto-ID dye showed that autophagy induction by cynaropicrin was significantly blocked in the presence of SB203580 (Fig. 7A and 7B). Consistent with this result, fluorescence microscopy showed that the expression of cynaropicrin-induced LC3 was significantly suppressed by SB203580 pretreatment (Fig. 7C). Additionally, the mitophagy-associated marker proteins PINK1 and Parkin in the mitochondrial fraction were reduced by SB203580 in cynaropicrin-treated cells, suggesting that p38 MAPK acts as an upstream regulator of cynaropicrin-induced autophagy and mitophagy (Fig. 7D). Furthermore, the phosphorylation of p38 MAPK by cynaropicrin was markedly inhibited in the presence of NAC (Fig. 7E), indicating that cynaropicrin-induced p38 MAPK activation was ROS-dependent.

## Discussion

Among the diverse pharmacological effects of cynaropicrin, it exerts antitumor and cytotoxic effects in different cancer cell lines. Although cynaropicrin exerts a cytoprotective effect by blocking the production of ROS caused by oxidative stress in normal cells [6, 9, 18], oxidative stress is recognized as a major factor that induces apoptosis in cancer cells [12, 13, 15, 16]. In addition, it was revealed that the induction of apoptosis and senescence by cynaropicrin in glioblastoma cells is accompanied by autophagy, and that ROS play a key role as regulators [12]. Cynaropicrin also induces autophagy in neuroblastoma, which is closely linked to ER stress and acts as an apoptosis inhibitor [11]. However, little is known about the relationship between cynaropicrin-induced autophagy and mitochondrial damage, which play a key role in inducing apoptosis.

Autophagy, an intracellular degradative process, is mediated by the formation of vesicles containing damaged organelles, protein aggregates, which further fuse with lysosomes to degrade and recycle the cellular contents within the vesicles [24, 34]. Autophagy is generally considered a survival process that maintains cellular homeostasis; however, it is also involved in cell death in the pathogenesis of many diseases [25, 35]. This autophagic cell death is associated with numerous physiological processes, including tumor suppression, and can activate other apoptotic signaling pathways [24, 25]. In our study, the formation of autophagy-specific vacuoles and the frequency of cyto-ID-positive cells were increased in HCC cells treated with cynaropicrin, indicating that autophagy is closely linked to cynaropicrin-induced cytotoxicity. In addition, the expression of LC3, an autophagic marker protein, was notably upregulated, as was the expression of LC3 II, a converted form of LC3 I that is involved in the formation of autophagosomes by tightly binding to the autophagosome membrane to induce autophagy [36, 37]. However, cynaropicrin-induced changes were not observed in THLE-2 cells, an immortalized human adult liver epithelial cell line. Unlike in neuroblastoma cells [11], the expression of Beclin-1and p62 were suppressed by cynaropicrin. In contrast, in glioblastoma cells, p62 expression is reduced upon induction of autophagy by cynaropicrin [12]. However, under conditions where cynaropicrin induced cytotoxicity in multiple myeloma cells, the expression of these proteins did not change, and autophagy was not observed [10]. Therefore, the roles of Beclin-1 and p62 in cynaropicrin-induced autophagy need to be redefined depending on the cancer cell line used.

Similar to the results of previous studies [10, 12], in the present study, cynaropicrin-induced cytotoxicity in HCC cells was closely associated with mitochondrial dysfunction through loss of MMP, an indicator of mitochondrial damage. The fact that cytochrome c is expressed predominantly in the cytoplasm rather than in the mitochondria suggests that the loss of MMP is induced. Mitophagy, a type of selective autophagy, is an intracellular decomposition mechanism that removes damaged or unnecessary mitochondria [38, 39]. When mitochondrial damage occurs, they are surrounded by a membrane to form autophagosomes that fuse with lysosomes to selectively remove the damaged mitochondria [40, 41]. Under conditions in which normal MMP is maintained, rapid degradation of the PINK1 protein occurs, and Parkin is present in the protoplasm. However, when mitochondrial damage occurs and MMP decreases, PINK1 is stabilized through autophosphorylation, the movement of Parkin to the mitochondria, and ubiquitin E3 ligase enzyme activity increases, thereby inducing mitophagy [42, 43]. Therefore, the cross-staining results between MitoTracker, LC3, and Cyto-ID in cynaropicrin-treated HCC cells demonstrated autophagosome formation. PINK1 and Parkin were predominantly expressed in the mitochondrial fraction, indicating that mitophagy was induced. However, pretreatment with bafilomycin A1 inhibited cynaropicrin-induced PARP cleavage and conversion to LC3 II, and decreased Beclin-1 expression, maintaining its expression at the control level. Furthermore, bafilomycin A1 blocked the cynaropicrin-induced translocation of PINK1 and Parkin to the mitochondria and abolished autophagy. These findings clearly demonstrate that the inhibition of proliferation and induction of apoptosis in HCC cells by cynaropicrin are mediated by mitophagy.

Mitochondria are the main ROS-generating organelles in cells, and are vulnerable to ROS. ROS act as signaling molecules that regulate autophagy; mitophagy is an essential mitochondrial protection mechanism, including the removal of damaged mitochondria, which is the main cause of mtROS generation [41, 44]. In Hep3B cells, cynaropicrin strongly induced ROS production starting at 30 min of treatment and completely blocked ROS production in the presence of NAC. mtROS production was also significantly increased in cynaropicrin-treated cells and was reduced to control levels by Mito-Tempo. In addition, when the production of ROS was artificially blocked, the fragmentation of PARP and the reduced cell survival rate caused by cynaropicrin were restored to control levels, which supports the previous finding that apoptosis of cancer cells induced by cynaropicrin was ROS-dependent [12, 16]. Moreover, the expression of mitophagy inducers, such as PINK1 and Parkin, and autophagy marker proteins, including LC3 and Beclin-1, which was altered by cynaropicrin, were maintained at the control level, indicating that mtROS acted as a key factor in the induction of mitochondrial autophagy by cynaropicrin in HCC cells. Although it has been suggested that inactivation of ERK among MAPKs may be involved in the anticancer activity of cynaropicrin [12, 15, 18], its role in apoptosis is unclear, and the involvement of JNK or p38 MAPK in cancer cell apoptosis has not been investigated. Contrary to these results, in this study, ERK phosphorylation increased at the beginning of cynaropicrin treatment, but the ERK inhibitor did not have a significant effect on the cytotoxicity caused by cynaropicrin treatment using Hep3B cells. In contrast, the p38 MAPK inhibitor notably counteracted the cytotoxicity and mitochondrial superoxide production induced by cynaropicrin. Furthermore, autophagy and increased expression of mitophagy players, such as PINK and Parkin, by cynaropicrin treatment was inhibited by the p38 MAPK inhibitor, and when mtROS production was blocked, phosphorylation of p38 MAPK was not induced. These findings imply that the excessive accumulation of mtROS induced by cynaropicrin in Hep3B cells may increase mitophagy and mtROS-dependent p38 MAPK activation. Recent studies have shown that the removal of damaged mitochondria due to mitochondrial oxidative stress is achieved through the regulation of the PINK and Parkin signaling pathways [45, 46]. These proteins accumulate in the endoplasmic reticulum (ER)-mitochondrial contact regions and regulate their crosstalk. Alterations in ER-mitochondrial tethering are recognized as a hallmark of mitophagy induction. This process is modulated by Ca^2+^ signaling [47, 48]. A recent study by Yang *et al*. [11] indicated that ER stress-mediated protective autophagy is involved in cynaropicrin-induced apoptosis of neuroblastoma cells. However, to date, the correlation between Ca^2+^-mediated ER stress and mitophagy has not been studied, and is worth exploring.

**Fig. 8:**
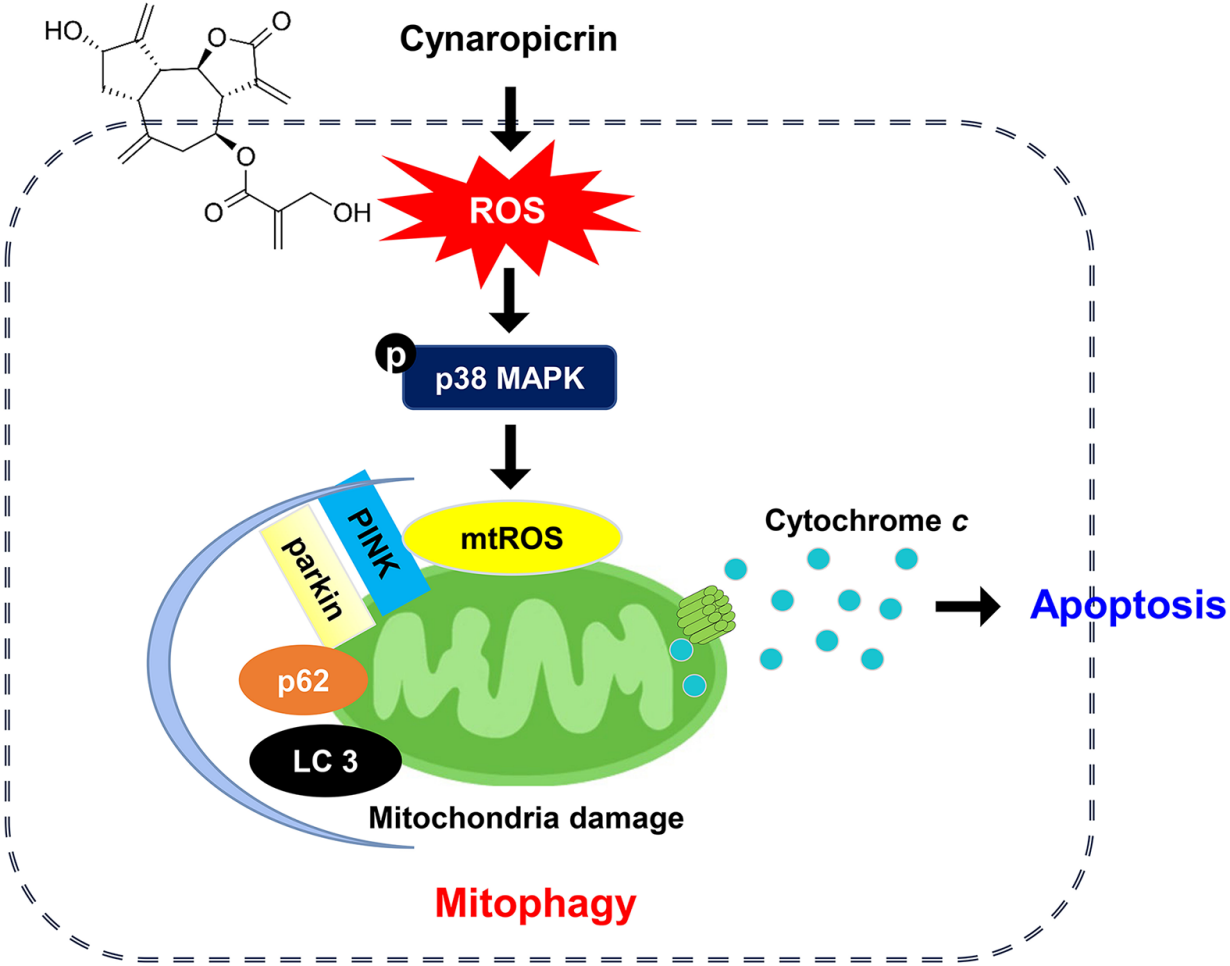
Schematic diagram showing the induction of cytotoxicity by cynaropicrin in Hep3B cells.

In this study, we explored the potential therapeutic effects of cynaropicrin in HCC cells and investigated its mechanism of action, particularly its role in mitochondrial autophagy. Our results showed that cynaropicrin cytotoxicity in HCC cells is associated with ROS-dependent mitochondrial dysfunction, apoptosis, and autophagy induction. In addition, cynaropicrin-induced increase in mtROS levels contributed to the promotion of mitochondrial autophagy, suggesting that ROS triggered cynaropicrin-induced autophagic apoptosis. Moreover, we demonstrated that mtROS-mediated p38 MAPK activation by cynaropicrin contributes to the regulation of protective mitophagy induction, as summarized in Fig. 8. In conclusion, our findings indicate that cynaropicrin can induce ROS-dependent autophagic apoptosis in HCC cells by enhancing mitophagy, and may be a potential therapeutic candidate for HCC.

## Supplemental Materials

Supplementary data for this paper are available on-line only at http://jmb.or.kr.
