# Supplementary material for: Cynaropicrin Suppresses Cell Proliferation by Inducing Mitophagy through p38 MAPK-Mediated Mitochondrial ROS Generation in Human Hepatocellular Carcinoma Cells
Source: J Microbiol Biotechnol. 2025 Apr 24;35:e2501025. doi: 10.4014/jmb.2501.01025 (PMC12089954; doi:10.4014/jmb.2501.01025)
Supplement: Supplementary file 1 [file jmb-35-e2501025-supple.pdf]

J. Microbiol. Biotechnol. 2025. 35: e2501025
https://doi.org/10.4014/jmb.2501.01025

Supplementary results

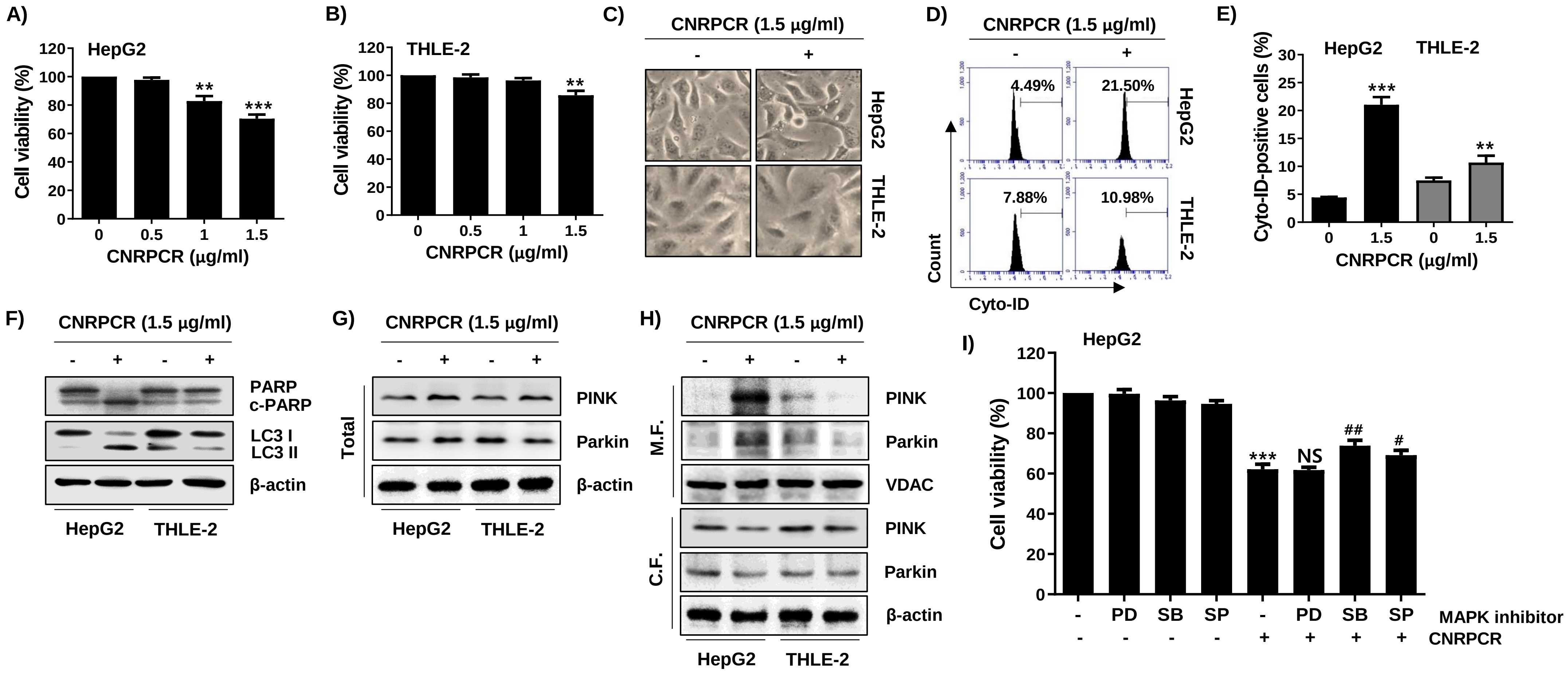

(A and B) The cell viability of HepG2 and THLE-2 cells cultured for 24 h in media containing various concentrations of cynaropicrin was investigated by MTT assay. (C-H) HepG2 and THLE-2 cells were treated with 1.5 µg/ml cynaropicrin for 24 h. (C) After treatment with cynaropicrin, changes in cell morphology were observed using phase contrast microscopy. (D and E) To investigate whether autophagy was induced in the two cell lines treated with cynaropicrin, flow cytometry was performed after Cyto-ID staining (D), and the results of flow cytometry were quantified and displayed as a bar graph (E). (F and G) The expression levels of key biomarker proteins of apoptosis, autophagy, and mitophagy were assessed by Western blotting using total cellular proteins isolated from cells after cynaropicrin treatment. (H) Mitochondria (M.F.) and cytosolic fractions (C.F.) were separated and the expression changes of mitophagy-regulating proteins PINK1 and Parkin were examined by Western blotting. (I) HepG2 cells were treated with either MAPK inhibitors, PD (PD98059, ERK inhibitor), SB (SB203580, p38 MAPK inhibitor) or SP (SP600125, JNK inhibitor), for 1 h and then stimulated with cynaropicrin (1.5 µg/mL) for 24 h. After treatment, cell viability was measured using the MTT assay.
